# Levels of Physical Activity and Psychological Well-Being in Non-Athletes and Martial Art Athletes during the COVID-19 Pandemic

**DOI:** 10.3390/ijerph19074004

**Published:** 2022-03-28

**Authors:** Armando Monterrosa Quintero, Ana Rita Echeverri Rios, Juan Pedro Fuentes-Garcia, Juan Carlos Gonzalez Sanchez

**Affiliations:** 1 Research Group SER-SICIDE, Department of Physical Education and Sports, Universidad Católica de Oriente, Rionegro 054040, Antioquia, Colombia; amonterrosa@uco.edu.co; 2 Faculty of Education Sciences, Universidad Católica de Oriente, Rionegro 054040, Antioquia, Colombia; ana.echeverri3007@uco.net.co (A.R.E.R.); jcgonzalez@uco.edu.co (J.C.G.S.); 3 Didactic and Behavioral Analysis of Sports Research Group (ADICODE), Faculty of Sport Sciences, University of Extremadura, 10003 Cáceres, Spain

**Keywords:** physical activity, lockdown, COVID-19, psychological well-being

## Abstract

Background: The objectives of this study were to identify which of the sociodemographic variables affected psychological well-being in two populations that differed in their sports practice at the end of the COVID-19 lockdown in Colombia. Methods: The study was conducted through an online survey using the IPAQ-S and PGWBI-S questionnaires six months after the first SARS-CoV-2 lockdown, between 8 and 22 September 2020 in Colombia. The total number of study participants was 582, subdivided into two groups: (i) non-athlete university population (NA) without constant practice in physical activity or sports training (*n* = 470); and (ii) martial arts athletes (*n* = 122) with a sports career (A), 7.4 ± 3.4 years of experience, at different levels (advanced belts and black belts). Results: Sports practice, normal BMI levels and high levels of physical activity translated into absence of distress (ND) in the psychological well-being of populations. The variance between the factors could be explained by the general health dimension (2.4% population; 4.2% sex; 12% physical activity; 2.6% age). A moderate correlation between vitality and MET was found (r = 0.33; *p* < 0.001). Conclusions: The practice of a sport such as martial arts, normal body mass index and high levels of physical activity were factors that positively reduced levels of distress, translated into better psychological well-being in populations, and the general health dimension presented important contributions to psychological well-being. Intervention plans must be carried out, especially in populations that do not practice physical activity—mainly female and those under 40 years of age.

## 1. Introduction

The lockdown policies of the SARS-CoV-2 pandemic (COVID-19) have affected the general population in different areas of life, notably by significantly decreasing levels of physical activity [1] and deteriorating psychological well-being [2]. These variables can be evaluated using online forms, an alternative that allows for greater safety due to the physical distance between people [3].

The literature has clearly argued that quarantine and social distancing measures are paramount in reducing the number of infections among populations, but along with these restrictions, negative consequences have arisen in psychological, health, and economic aspects [3,4]. Social distancing has caused important changes in the psychological well-being of people, especially females, who have shown higher levels of stress when compared to males, an aspect that translates into an increase in confusion and anxiety [5,6]. Regarding mental health, experts have mentioned that because of the emerging situation, there has been an increase in anxiety and stress, which results in post-traumatic stress, confusion, anger, emotional disorders, low mood, irritability, and insomnia [7,8]. Similarly, the lockdown policies led to a decrease in physical activity which was reflected in very well-established sequelae by studies where adverse effects were prominent in health, highlighting premature aging, obesity, cardiovascular vulnerability, muscle atrophy, bone loss, and decreased aerobic capacity [3].

In addition, the literature mentioned that physical inactivity and the lockdown measures had negative consequences on the emotional factors of people and athletes [1,2] in contrast to physical activity and physical exercise on a scheduled basis, which confirmed the hypothesis of bodily well-being and comprehensive health through these strategies [3].

Hypothetically, physical activity seems to have neural and biological effects on people, especially via behavioral factors such as emotional stability and in the treatment of mental disorders [9], although the practice of moderate exercise at levels not higher than 60% (VO2Max) can be achieved in exercise periods lasting 20 min a day, depending on the capacity and emotional state of individuals [5,6]. 

Published research devoted to studying the effects of lockdown by SARS-CoV-2 reviewed to date suggests varied benefits of physical activity and exercise in different populations, including sedentary individuals, as well as athletes of different skill levels [10,11], observing that high-intensity interval training (HIIT) and moderate-intensity training decreased anxiety, stress, and depression, and increased resilience in healthy adults during the COVID-19 pandemic [10]. 

At the individual level, alterations have been evidenced in habitual practices, work, interpersonal relations, health and leisure, and sports, among others, which affect the physical and psychological health of people [5,6,9]. Several publications have reported the interest in this topic by researchers who have studied SARS-CoV-2 in athletes from different sports disciplines: Olympic and Paralympic [12] elites and semi-elites [13,14,15]; athletes [16]; handball players [17]; chess players [18]; or martial arts [11].

According to global public health data, physical activity levels were considered a widespread problem due to the lack of practice before the COVID-19 pandemic [12]. In Latin America, especially in Colombia, citizens between 18 and 64 years old were inactive according to the guidelines from the World Health Organization. The Mandatory Preventive Isolation policies in Latin America, especially in Colombia, were some of the measures of physical and social distancing imposed during the COVID-19 pandemic. These measures, due to the seriousness of the impact of the pandemic on public health, had to be implemented at the widespread social and economic levels, and affected the daily activities of citizens [19]. The objective of the Colombian government’s measures aimed at containing the spread of the virus, and due to these circumstances, confinements and controls were decreed as of 25 March 2020, beginning with a period of 19 days of confinement [19]. On 17 April, the new measures called “Collaborative and Intelligent mandatory Preventive Isolation” were reported in a new stage of the quarantine [20]. As of 4 May 2020, work was allowed in some sectors of industry as a measure to avoid economic crises due to the pandemic [21]. As of 1 September, the entire country opened, without restrictions, and airports were also reopened to travelers, except those with symptoms of COVID-19 or their relatives. In addition to the restrictions imposed by the national government, some local governments imposed other restrictions. These consisted of commercial, banking or notarial activities, and only those whose national identity document number ended in even or odd digits could leave their homes [21].

In our opinion, the different studies carried out when comparing populations of athletes with non-athletes may be inconsistent due to cultural, social, time period, and economic variables that are specific to each region [22,23]. Due to the aforementioned issues, this study aimed to identify which sociodemographic variables (athletic level, sex, BMI, level of physical activity, and age category) affected the psychological well-being by comparing two groups that differed in their practice of sports: martial arts athletes (A) and non-athletes (NA) at the end of the strict lockdown in Colombia.

## 2. Materials and Methods

### 2.1. Procedures

Non-athlete participants were recruited by distributing an invitation through administrative channels at the Universidad Católica de Oriente (UCO) through social media (WhatsApp, Version 2.19.368 Classroom, and institutional email, Meta Platforms, Inc., Menlo Park, CA, USA). A standardized questionnaire was developed with Office 365^®^ (Microsoft Office Professional plus, 2019, version 1808, Microsoft Corporation, Albuquerque, NM, USA), including validated tools to assess physical activity (PA; i.e., the International Physical Activity Questionnaire–Short Form (IPAQ-SF) in Spanish, which contained seven questions and was designed to assess changes in PA) and mental well-being (i.e., the Psychological General Well-Being Index-Short version (PGWBI-S)) [24]. The online survey was sent to potential respondents (employees and students) and was accessible via an anonymous link distributed via email. The survey was announced by the Human Resources (HR) department and physical education faculty and it was open over a period of 14 days during the COVID-19 emergency in Colombia. Automated reminders were sent two times during this time period. A brief introduction section prior to the different questionnaires explained the objective of the survey.

Data were collected in 2020 from 8 to 22 September via an online survey. After having signed an informed consent form, the participants completed a web-based survey which was launched online at https://tinyurl.com/ybsrz9z5 (accessed on 30 September 2020). The self-administered survey remained online for 14 days and could be completed in approximately 10–15 min. It included the IPAQ-SF and PGWBI-S questionnaires, together with questions related to demographic and socioeconomic data. 

The athletes were initially recruited by phone, email, WhatsApp, or using our informal and professional networks. They were informed about the study purpose and methodology. Before starting the survey, it was reiterated that it would be completely anonymous and not traceable to the identity of the participants. In addition, the athletes were advised on the assessments performed to avoid the learning effect. Subsequently, the results of the analyzed athletes were classified according to their sociodemographic level.

### 2.2. Participants’ Study Design and Study Population

This study was conducted through an online survey six months after the first SARS-CoV-2 confinement between 8 and 22 September 2020, in Colombia. The total number of study participants was 582, subdivided into two groups: (i) people from the university population (non-athletes, NA) without constant practice in physical activity or sports training (*n* = 470); and (ii) martial arts athletes (*n* = 122) with a sports career (A) of 7.4 ± 3.4 years of experience, at different levels (advanced belts and black belts). The study was approved by the ethics committee of the Universidad Católica de Oriente through Resolution 8430/1993 of the Ministry of Health and Social Protection of Colombia together with the bioethics and biosafety commission of the University of Extremadura (Spain) approval number 57/2020, following the Declaration of Helsinki. The online data collection was performed using the same questions in the form of Microsoft Forms, Office 365^®^ (Microsoft Office Professional plus 2019 for NA and Google Forms^®^ (Google LLC, Mountain View, CA, USA) for A.

### 2.3. Sociodemographic Variables

After having read and approved the informed consent form, the following sociodemographic variables were collected: age (years); height (cm); weight (kg); body mass index (BMI, kg·m^−1^); and sex (male and female). For both athletes and non-athletes, the same variables were collected by the online forms, along with the questions related to levels of physical activity and psychological well-being. The characteristics of the sociodemographic variables can be observed in Table 1.

### 2.4. Measures

#### 2.4.1. International Physical Activity Questionnaire—Short (IPAQ-S)

Monitoring physical activity levels during a lockdown may be interesting for public health. A good instrument for investigating the physical activity rate in a large population is the International Physical Activity Questionnaire (IPAQ). This instrument was developed by various researchers from different countries with the support from the World Health Organization (WHO) and the US Centers for Disease Control and Prevention (CDC) [25] in the short form of seven items [26]. IPAQ’s unique feature is that it assesses all believed health-related physical activities that can take place in different settings, and it has already been used, among other cases, to analyze the COVID-19 nationwide lockdown and physical activity profiles among the Northwestern Italian population [17]; and the Spanish version has also been validated and applied in different studies, such as in the evaluation of the metabolic equivalent of tasks (METs) of the participants [27] 

The IPAQ questionnaire (http://www.ipaq.ki.se, (accessed on 15 August 2020) was developed to assess self-reported physical activity levels in adults aged 18–79 [15]. It focuses on collecting information about the duration, frequency, and intensity of physical activity in four domains: (1) work; (2) transport; (3) domestic and gardening; and (4) leisure time during the last 7 days, so it allows evaluating different levels of physical activity during the lockdown due to COVID-19, providing information on the minutes per day and days per week at any time, with these values transformed into a metabolic equivalent of tasks (METs). The information captured in the questionnaire reports various types of physical activity (low, moderate, and very active), classified as walking activities and the time spent sitting on an ordinary day, including the working days, moderate activities (i.e., carrying light loads and cycling at a regular pace, exercising in the garden); and strong activities (i.e., heavy lifting, intense aerobic exercise, cycling and/or treadmilling) [3].

For the evaluation of physical activity levels, the recommendations were taken by the IPAQ, where the values of low activity (<600 MET-min/week); moderately active (600 MET-min/week); and very active (3000 MET-min/week) were the means of comparison between participants [28].

#### 2.4.2. Survey for Mental Well-Being Measurement

To assess psychological well-being (levels of distress) in its dimensions of anxiety, vitality, deep depression, self-confidence, and positive well-being, the General Psychological Well-Being Index, abbreviated “PGWBI-S”, was used [24]. The questionnaire is composed of six items with six possible answers with values from 0 to 5, for a total value of 30 points. The sum of the values of the six dimensions is considered the total score. For the similarity between the original version and the short version of the PGWBI, each value from each response was multiplied by 3.66. The total score was compared with a six-item scale in which values less than 60 reflected severe distress; scores between 60 and 69 defined moderate distress; scores between 70 and 89 meant without distress; and values greater than 90 indicates a positive well-being status. For the reliability analysis of the instrument, Cronbach’s Alpha was used, yielding a value of 0.87, which indicated a good validity of the data [29].

#### 2.4.3. Statistical Analysis

All analyses were performed using the Jamovi Software^®^ version 1.6 (https://www.jamovi.org, accessed on 15 December 2021). Sociodemographic data and results are presented as percentages for qualitative variables through contingency tables and as the mean ± standard deviation for quantitative variables. The normality distribution of the data was evaluated with the Kolmogorov–Smirnov test, and the homogeneity of variance was verified with the Levene’s test. The interaction between the variables was analyzed with an analysis of variance (ANOVA) test with a mixed component, and Tukey’s post hoc tests. A multivariate analysis of variance (MANOVA) was performed to evaluate the different dimensions that shape the PGWBI. Associated univariate comparisons between groups were performed using Spearman’s correlation. The internal consistency of the PGWBI instrument was corroborated the Cronbach’s Alpha test (α) having a value greater than 0.7 meets the homogeneity budget within the scale of the instrument [28]. A *p* ≤ 0.05 significance value was used for the entire statistical treatment, with a 95% confidence interval. 

The sample size for the NA group was calculated using the equation for finite populations [30] by means of a sample calculator (https://www.netquest.com/en/thanks-sample-calculator, accessed on 30 July 2020) with a universe population of 3600 people, using a margin of error of 5%, and a confidence interval of 95%. For the A group, the sample size was determined using the G *power program through a sample calculation (α = 0.05 and statistical power = 80%), based on effect sizes reported in previous studies [3]. For the A group, the following inclusion and exclusion criteria were considered:

I. Have more than 3 years of experience in sports practice.
II. Being older than 18 years old;
III. Being part of the Colombian Federation of Taekwondo;
IV. Not presenting serious sports injuries or having COVID-19 before or during the application of the questionnaire.

#### 2.4.4. Experimental Design

This study was created and carried out to obtain relevant information when comparing the levels of physical activity and sociodemographic variables: age (years); height (cm); weight (kg); body mass index (BMI, kg·m^−1^); and sex (male and female)—and how these affect the psychological well-being of the participants. The data of the participants were originally used for tabulation and statistical treatment, being sent by the researchers through the channels of the sports federation and the Universidad Católica de Oriente (UCO).

The compilation was carried out using the media of the university and the sports federation. At the time of application of the instrument, the population was confined due to policies of the Colombian government, which made it possible to evaluate the different factors of the study.

To develop the analysis of our research, we utilized a factorial design that was composed of five study variables:

I. Population factor, divided into two levels (athletes and non-athletes);
II. Sex factor, divided into two levels (male and female);
III. Body Mass Index Factor, subdivided into three levels (low, normal, and overweight)
IV. Physical Activity Factor, subdivided into three levels (low, moderate, and high);
V. Age category factor, subdivided into three levels (18 to 19 years old, 20 to 40 years old, and ˃41 years old).

The factors were first compared between the population groups (athletes and non-athletes) evaluating the different variables that shaped the other variables (Table 1).

Subsequently, the population groups were grouped using the sex of the participants as a reference, making comparisons with the other variables (Table 2).

Then, all the factors were compared with the dimensions of psychological well-being to searching for significant differences between the variables, and in turn, add them to determine the psychological well-being index (Table 3 and Table 4).

## 3. Results

### 3.1. Descriptive Data of the Population by Sex and Comparisons between the Study Variables

The comparison between the general characteristics with sex and the effect of the sport was calculated using the ANOVA test and Tukey’s post hoc tests (Table 2). The analysis of the sociodemographic variables, when compared with the dimensions of psychological well-being, showed significant differences between the groups, identified by means of the MANOVA (F = 8.51; *p* ˂ 0.001) observed in Table 3.

The findings showed significant differences between the populations (A and NA) according to sex when compared with the general characteristics (Table 2). Tukey’s post hoc tests revealed age differences in males according to the studied populations (A and NA). The athletes tended to be older (F = 11.4; *p* ˂ 0.05); in the ˃41 years age category, the athletes were older than the non-athletes (F = 3.5; *p* ˂ 0.05).

Each of the dimensions that comprise the PGWBI (6) were compared with the different factors using the MANOVA test and Tukey’s post hoc tests (Table 3). The results showed significant differences between the populations in terms of the dimensions of vitality, positive well-being and general health; 2.4% of the highest variance ratio can be explained by general health in the population factor (F = 14.6; *p* ˂ 0.01; η²*p* = 0.024). In the sex factor, significant differences were found in all dimensions, where 4.2% of the variance can be explained by the General Health dimension (F = 25.6; *p* ˂ 0.01; η²*p* = 0.042). When verifying the effect of physical activity on psychological well-being, differences were found in anxiety, depression, self-control, positive well-being, and general health, finding the highest value of vitality, which explained 12% of the variance in the effect of physical activity levels. (F = 40.1; *p* ˂ 0.001; η²*p* = 0.120). The effect of the age factor was significant in all dimensions in which 2.6% of the variance explained the effect of age through general health (F = 7.3; *p* ˂ 0.01; η²*p* = 0.026) [30].

The results of the two-way ANOVA (Tukey’s post hoc tests) did not show significant differences for the sex vs. groups and sex vs. age when comparing all the dimensions that comprised the psychological well-being in our study. When summarizing the different dimensions of psychological well-being, a greater degree of distress was established among females than males, regardless of whether they were athletes or non-athletes.

### 3.2. Correlations between the Study Variables and the Dimensions of Psychological Well-Being

Small significant correlations (r = 0.1 to 0.3) were found between age, METs, and all dimensions of psychological well-being (Table 5), where vitality presented a moderate correlation (r = 0.3–0.5) according to the Hopkins’ scale [31].

## 4. Discussion

In our opinion, this study is a pioneer in comparing people who do not practice sports with experienced martial arts athletes at the end of strict confinement in Colombia. The confinement measures decreed by governments due to SARS-CoV-2 (COVID-19) produced structural changes at the physical and psychological level in the general population, where athletes from different sports disciplines and non-athletes were severely affected due to the measures implemented to minimize infections worldwide.

### 4.1. Impact of Lockdown on Physical Activity and Psychological Well-Being

The literature reports physical and psychological health problems in people who are in long periods of lockdown [11] due to the lack of physical exercise, sociability, and recreation [3,32]. However, despite the ability of athletes to endure and control stress levels, they may present levels of anxiety and depression, as shown by the results found when comparing the different sociodemographic variables, psychological dimensions, and physical activity in our study in the two groups of participants [33].

The results found indicate that a lockdown negatively affects levels of physical activity, as shown by the deterioration of psychological well-being, especially in females, where more inactivity than males was found in other studies (57.4% vs. 42.6%). These findings are in agreement with our results, and it is believed that this is due to psychosocial and cultural factors [34]. Likewise, our study seems to agree with this trend during the lockdown, and in turn, we have found significant differences in the level of METs between the groups, which allows us to deduce that despite the lockdown, the A maintained a higher level of METs than the NA, which suggests a greater physical and psychological stability [35].

Some studies with samples larger than ours across 122 countries reported that 31.1% reported physical inactivity among the population, below the parameters of normality established worldwide [26]. Similarly, another study carried out with a similar methodology but with an NA population showed that the research participants were in physical inactivity with values between 44% and 50%, which places them between moderate and very active [36].

When comparing the factors with the six dimensions of psychological well-being (Table 3), we noted that the A group, due to their training levels, had higher values in the positive aspects, which translated into lower levels of anguish. Regarding the sex differences, a higher level of distress was found among females, which could suggest that they were more affected by the pandemic; however, it is important to clarify that there is no knowledge of their previous mental health conditions, so we believe these statements should be taken with caution, encouraging more studies in this regard in female athletes [37,38,39].

Nevertheless, despite the fact that the results showed a higher rate of psychological well-being in those who performed physical activity, the decrease in training or physical exercise is associated with an unfavorable or lower state in the perception of well-being, which is highly important. Similar trends were found in another study, despite the fact that the populations differed in the amount of training and sports level [3]. The above follows from the fact that physical activity, or training for athletes, requires discipline and continuous practice, which was not possible during the health restrictions, resulting in the sensation of a decrease in health and well-being conditions in general. Although the effects of these conditions may take some time, the feeling of loss is largely due to the subjective assessment of what has been built with dedication [22,40].

Analyzing our results and comparing them with the literature, we noted that high-intensity levels confirmed that a higher level of training indicated a better psychological well-being, especially in vitality, where moderate correlations were found between study participants. The general health dimension seems to be an axis between the different factors, as it ranked first in the percentages of explanation of the variance found among the variables in our study.

### 4.2. Psychological Well-Being and Lockdown

The lockdown measures imposed by all states greatly helped to contain SARS-CoV-2 virus infection [41], but in turn, negative sequelae were observed in different communities, including the A population, whom, due to their social situation, live in solitude, where levels of depression and other types of mental illness can lead to a lower quality of life due to physical and mental deterioration. This suggests that different psychological dimensions may be affected by lockdowns, and thus projected in anger and hostility, especially in young adults [42,43,44].

The psychological factor seemed to be one of the most affected by distancing policies in populations, especially in the area of sports, where high levels of anxiety and depression were reported in females [44], in whom a higher stress score was observed [1]. In fact, in our study, we found more significant correlations between physical activity levels and psychological dimensions, with higher values in the scores in the total sum of the dimensions, in which stress prevailed in females, with results similar to those of other studies [15,16,45].

The A group is exposed to strenuous training, the objectives of the competition, and the eagerness to win or the fear of losing, and among other more subjective factors, we also find the ability to recover in the face of defeat, the sporting individuality, which is an important factor in victory, and the very renowned resilience [46]. Although these factors can positively affect their physical and psychological integrity, A do not always escape lockdown sequelae, as shown in different studies [47,48].

Our results showed that the decrease in physical activity, training, or physical exercise was related to an unfavorable state at the level of psychological well-being, and showed results resembling the trends reported in other studies, although populations differed in the amount of training and sports level [3]. It is considered necessary to take into account the psychological and stress aspects within athletes due to their high preparation load, as they can also be victims of their training systems when they are not performing any physical activity. This points to the scenario of the lockdown by the pandemic as an opportunity to design and apply research in new ways to face adverse situations in pandemic lockdowns, using different methods promote mental health well-being, especially in sports performance, taking into account that A are considered examples in society [14].

The correlations found in our study between the levels of physical activity and the dimensions indicate that greater physical activity indicates better levels of vitality (r = 0.33; *p* ˂ 0.001) and general health (r = 0.26; *p* ˂ 0.001), which translates into optimal well-being among populations that perform arduous and constant physical activity [49].

Our study compared some general factors such as sex, and the findings showed that the female sex had greater stress, and when comparing people who performed sports, differences were observed especially in the dimensions of positive well-being and general health, with more benefits observed in the A group, in which levels of anguish were not observed (*p* < 0.01). When analyzing the nutritional category, differences were found in BMI levels, with lower stress values observed among participants who had normal values (18.5–24.9). The physical activity that people performed during the pandemic also presented low levels of stress in people who performed intense activity, especially the A group. Our hypothesis referred to the adaptation that athletes have, which reflects how physical exercise influences psychological well-being. Regarding the age category, people over 41 years of age had lower levels of anguish. We considered that this behavior was due to the lack of monetary stability of those who were younger, which translates into economic tension and less purchasing power [50], and we also believe that the results were not very encouraging in female athletes, whereas males showed a greater perception of psychological well-being with higher scores in all scales and females presented moderate distress regardless of whether they practiced any physical activity. These results are supported by other studies, among which we highlight that of [51], which found a greater affective negativity in females and greater affective intensity in general than males with the emotional load being an important factor when evaluating the present situations. This may explain why the crisis that arose from the effects of lockdown generated higher levels of stress and anxiety in females [52]. Another factor that we considered important, was the role assigned to the female sex in terms of caregiving for others tasks, which could lead to greater emotional responsibility and a greater experience of emotions [53,54].

Among the strengths, we highlight the size of the sample, the easy application of the instrument in the country, the support from sports establishments at the departmental and national level, as well as the ease of obtaining the results due to the online forms utilized. Another aspect to highlight is the originality of this study, because the application of this methodology in A population compared to NA at the national level at the end of strict confinement for COVID-19 in Colombia was not found in the literature.

## 5. Limitations

Our findings may contain several limitations that should be discussed and considered for future research. First, the nature of the study, which is a cross-sectional and self-reported survey, has certain weaknesses, as it may prevent the detection of a possible bias in the physical activity measures (during confinement). Although the volume of physical exercise of each participant in the study was obtained from standardized questionnaires, in the absence of physiological measures of physical condition, we cannot rule out some inaccuracies in the time or intensity of the exercise performed. Second, the sample of athletes was limited and may have introduced bias. Additionally, the findings from these populations may not necessarily apply to the general public or to other communities that may be of lower socioeconomic, physical, or educational backgrounds.

Within our study, the results may be biased in the sampling due to the variability of the information, especially in the methodological limitations and strength of the study participants. Sex, the practice of sports, and the level of physical activity play an important role in obtaining truthful information when administering the questionnaire, and therefore, we noticed a greater number of participants who did not practice sports (79.6%), a situation that may have influenced the analysis due to the variability of the data [55,56]. However, these results should be interpreted with caution due to the context of today’s society. On the other hand, a few studies of the found, especially with this type of variable and study populations, hinder the discussion, especially when comparing results between the researchers.

Another limitation is that concerned with the bias in the sampling due to the variability of the information, especially in the limitations and methodological soundness of the study participants. Sex, BMI levels, sports practice, and physical activity level play an important role in obtaining truthful information when answering the instrument; therefore, we noticed a greater number of participants who do not practice sports, a situation that can influence the analysis due to the variability of the data, however, these results should be interpreted with caution due to the context of today’s society. On the other hand, the scarce literature on studies such as ours allows less objectivity when discussing results in this type of population and the current situation due to the lack of comparisons between the main variables of our study.

## 6. Practical Applications

The information obtained through this study allows us to show the impact on of lockdown measures on psychological well-being in the study populations, especially in females. We recognize that the effects of lockdown can have lasting effects; therefore, we encourage building psychological intervention policies and establishing measures where people are re-adapting to the new situations present at the global level, in which governments and sports entities must develop and support [13].

## 7. Conclusions

The findings of our study allow us to establish the hypothesis that sports practice—especially martial arts—normal body mass index, and high levels of physical activity, are factors that positively reduce levels of distress and translate into better psychological well-being in the populations studied herein. On the other hand, our study suggests that intervention plans should be carried out, especially in populations that do not practice physical activity, specifically females and those under 40 years of age. The effect of physical activity in populations was evidenced due to the correlation between METs and vitality, which translates into a better psychological well-being of individuals. The relevance of this research denotes the importance of physical activity in all types of populations during a pandemic, where females, in a greater proportion, may be disproportionately affected [1]. Therefore, new research is proposed in this type of population, where variables such as hormonal rates can be monitored to solve some gaps observed in our research.

## Figures and Tables

**Table 1:** Sociodemographic details of the non-athlete (*n* = 470) and athlete (*n* = 122) populations.

Morphological Information *	Non-Athletes	Athletes
Age (years)	28.8 ± 11.1 ^#^	33.7 ± 14.8 ^#^
Height (cm)	167 ± 20.7 ^#^	171 ± 8.5 ^#^
Weight (kg)	66.6 ± 13.5	69.6 ± 12.1
BMI (kg·m^−1^)	23.9 ± 3.93	23.9 ± 3.87
METs	2360 ± 2653 ^#^	3566 ± 2798 ^#^
**Sex**		
Male	209 (44.5%)	86 (70.5%)
Female	261 (55.5%)	36 (29.5%)
**BMI levels**		
Underweight	28 (6.0%)	9 (7.4%)
Normal weight	296 (63.0)	71 (58.2%)
Overweight	146 (31.1%)	42 (34.4)
**Physical activity level**		
Low active	156 (33.2%)	17 (13.9%)
Moderately active	165 (34.1%)	41 (33.6%)
Very active	149 (31.7%)	64 (52.5%)
**Category age**		
18 to 19 years old	87 (18.5%)	26 (21.3%)
20 to 40 years old	303 (64.5%)	56 (45.9%)
˃41 years old	80 (17%)	40 (32.8%)

* = values expressed as mean ± SD; ^#^ = *p* ≤ 0.05; BMI = body mass index; METs = metabolic equivalent of task.

**Table 2:** The description of the categorical results according to general characteristics, BMI level, physical activity level, and age category (comparison between sex and population).

Results	Female		Male	
	Athletes	Non-Athletes	Athletes	Non-Athletes
**General characteristics**				
Age (years)	26.5 ± 9.6	28.2 ± 11.2	36.8 ± 15.6 *	29.7 ± 10.9 * (ES 0.6)
Height (cm)	164 ± 8.7	160 ± 5.65	174 ± 6.41	174 ± 6.76
Weight (kg)	62.4 ± 10.7	60.3 ± 10.1	72.6 ± 11.4	74.6 ± 12.9
BMI	23.3 ± 4.0	23.3 ± 3.9	24.1 ± 3.8	24.6 ± 3.8
METs	3403 ± 3196	2082 ± 2419	3635 ± 2632	2707 ± 2888
**BMI levels**				
Underweight	16.9 ± 0.0	16 ± 4.4	17.8 ± 0.5	17.3 ± 1.0
Normal weight	22 ± 1.5	21.9 ± 1.6	21.8 ± 1.7	22.5 ± 1.6
Overweight	28.7 ± 5.0	28.4 ± 2.92	27.9 ± 2.4	28.3 ± 2.9
**Physical activity level**				
Low	368 ± 103	219 ± 189	270 ± 237	215 ± 201
Moderate	1632 ± 759	1455 ± 639	1860 ± 623	1655 ± 736
High	6548 ± 2986	5357 ± 2191	5326 ± 2104	5651 ± 2738
**Age category**				
18–19 years old	18.1 ± 0.7	18.2 ± 0.7	17.5 ± 1.6	18.2 ± 0.7
20–40 years old	26.3 ± 5.1	25.8 ± 5.8	29.1 ± 6.7	27.6 ± 6.2
˃41 years old	46 ± 3.7	49.9 ± 5.8	52.9 ± 8.0 *	47.8 ± 6.0 *(ES 0.8)

* *p* < 0.05, significant differences; ES = effect size.

**Table 3:** The results according to the PGWBI dimensions vs. study variable (population, sex, BMI level, physical activity level, and age category) according to the MANOVA test (Pillai’s Trace).

	Anxiety	Vitality	Depression	Self-Control	Positive Well-Being	General Health	PGWBI Score
**Population**							
Athletes	14.2 ± 4.4	11.8 ± 4.0 ^$^	12.2 ± 4.2	11.3 ± 5.4	10.5 ± 4.9 ^$^	11.9 ± 4.7 ^$^	ND
Non-athletes	13.2 ± 5.5	10.8 ± 4.4 ^$^	12 ± 4.8	11.8 ± 5.1	11.8 ± 4.5 ^$^	9.96 ± 4.9 ^$^	D
ES (between population)	-	0.23	-	-	0.27	0.38	-
**Sex**							
Female	12.4 ± 5.6 *	10.3 ± 4.3 *	11.6 ± 4.6 *	10.9 ± 5.1 *	11.1 ± 4.3 *	9.34 ± 4.8 *	D
Male	14.3 ± 4.7 *	11.7 ± 4.2 *	12.4 ± 4.7 *	12.4 ± 5.2 *	11.9 ± 4.8 *	11.4 ± 4.9 *	ND
ES (between sex)	0.36	0.32	0.17	0.28	0.17	0.41	-
**BMI levels**							
Underweight	11.8 ± 7.1	10.2 ± 4.6	11.8 ± 5.0	10.4 ± 5.7	10.6 ± 4.6	11 ± 5.2	D
Normal weight	13.5 ± 5.1	11.4 ± 4.1	12.1 ± 4.5	11.6 ± 5.1	11.5 ± 4.5	10.4 ± 4.9	ND
Overweight	13.2 ± 5.4	10.5 ± 4.6	11.9 ± 5.1	11.9 ± 5.4	11.7 ± 4.7	10.1 ± 5.1	D
**Physical activity levels**							
Low	11.9 ± 6.1 ^a^	9.63 ± 4.5 ^c^	11.3 ± 5.2 ^e^	11.2 ± 5.4 ^g^	10.8 ± 4.6 ^i^	9.44 ± 5.1 ^l^	D
ES (low vs. moderate)	0.24	-	-	-	-	-	-
Moderate	13.1 ± 5.1 ^ab^	10.2 ± 4.4 ^d^	11.5 ± 4.5 ^f^	11 ± 5.2 ^h^	10.9 ± 4.6 ^k^	9.58 ± 4.9 ^m^	D
ES (low vs. high)	0.57	0.82	0.37	0.29	0.41	0.49	-
Hight	14.8 ± 4.3 ^ac^	13 ± 3.3 ^cd^	13.1 ± 4.2 ^ef^	12.7 ± 4.95 ^gh^	12.7 ± 4.3 ^ik^	11.9 ± 4.5 ^lm^	ND
ES (moderate vs. high)	0.32	0.70	0.34	0.32	0.369	0.46	-
**Age category**							
18 to 19 years old	12.8 ± 5.2 ^A^	10.9 ± 3.9	11.4 ± 4.5 ^C^	10.8 ± 5.3 ^E^	11.1 ± 4.7 ^H^	10.1 ± 5.1 ^J^	D
ES (19 vs. 20–40 years old)	-	-	-	-	-	-	-
20 to 40 years old	13.1 ± 5.5 ^B^	10.7 ± 4.62 ^#^	11.7 ± 4.8 ^D^	11.5 ± 5.3 ^F^	11.3 ± 4.6 ^I^	9.92 ± 5.0 ^K^	D
ES (19 vs. 41 years old)	0.43	-	0.40	0.40	0.32	-	-
˃41 years old	14.7 ± 4.5 ^AB^	12.1 ± 3.8 ^#^	13.5 ± 4.1 ^CD^	13.1 ± 4.6 ^EF^	12.7 ± 4.1 ^HI^	11.9 ± 4.4 ^JK^	ND
ES (20 to 40 vs. 41 years old)	0.30	0.32	0.40	0.32	0.32	0.40	-

Note. * *p* < 0.05 significances differences; BMI = body mass index; ES = effect size; ND = no distress; D = distress; PGWBI-S = Psychological General Well-Being Index—Short; ^$^ = differences between populations; letters denote (lowercase letters “a,b,c,d,e,f,g,h,i,k,l,m”) = differences between very active and the other levels of physical activity in the different dimensions of the PGWBI-S; letters denote (capital letters “A,B,C,D,E,F,H,I,J,K”) = differences between >41 years and the other age category in the different dimensions of the PGWBI-S; ^#^ = differences between 20 to 40 years and >41 years.

**Table 4:** Comparison of the effect of sex vs. population and sex vs. age on the dimensions of psychological well-being.

		Anxiety	Vitality	Depression	Self-Control	Positive Well-Being	General Health	PGWBI Score
Female	Athletes	12.6 ± 4.3	10.1 ± 3.9	11.4 ± 4.0	9.7 ± 4.9	9.6 ± 4.6	10.1 ± 4.6	D
	Non-athletes	12.4 ± 5.8	10.4 ± 3.9	11.6 ± 4.3	11.1 ± 5.5	11.3 ± 4.3	9.2 ± 4.8	D
Male	Athletes	14.9 ± 4.4	12.6 ± 4.4	12.6 ± 4.7	11.9 ± 5.2	10.9 ± 5.0	12.6 ± 4.6	ND
	Non-athletes	14.1 ± 4.8	11.4 ± 4.3	12.4 ± 4.9	12.7 ± 5.0	12.4 ± 4.6	10.9 ± 5.0	ND
Female	18 to 19 years old	11.3 ± 5.6	10.5 ± 3.2	11.3 ± 4.7	9.2 ± 5.0	10.8 ± 4.4	9.4 ± 5.0	D
	20 to 40 years old	12.3 ± 5.6	10.1 ± 4.5	11.1 ± 4.6	10.7 ± 5.2	10.8 ± 4.4	8.9 ± 4.7	D
	˃41 years	14.0 ± 5.6	11.4 ± 4.3	14.1 ± 4.2	13.3 ± 4.5	13.1 ± 3.5	11.3 ± 4.4	ND
Male	18 to 19 years old	13.7 ± 4.9	12.3 ± 4.7	12.0 ± 4.7	12.9 ± 5.0	11.7 ± 5.2	11.6 ± 5.0	ND
	20 to 40 years old	14.1 ± 5	11.3 ± 4.4	12.2 ± 4.9	12.2 ± 5.3	11.8 ± 4.8	11.0 ± 5.1	ND
	˃41 years old	15.2 ± 3.2	12.5 ± 3.4	13.2 ± 4.1	13.0 ± 4.7	12.5 ± 4.4	12.3 ± 4.4	ND

Note: D = distress; ND = no distress.

**Table 5:** Correlations between study variables.

	Age	Body Mass	Height	BMI	METs
Age	1				
Body mass	0.318 ***	1			
Height (cm)	0.014	0.235 ***	1		
BMI	0.347 ***	0.819 ***		1	
METs	−0.024	0.029	0.031	−0.056	1
Anxiety	0.186 ***	0.053	−0.035	0.002	0.215 ***
Vitality	0.173 ***	0.047	−0.033	−0.009	0.335 ***
Depression	0.21 ***	0.045	0.035	−0.003	0.177 ***
Self-control	0.184 ***	0.093 *	0.105 *	0.016	0.135 **
Positive well-being	0.164 ***	0.069	−0.002	0.043	0.179 ***
General health	0.171 ***	0.068	0.087 *	−0.036	0.262 ***

Note. * *p* < 0.05, ** *p* < 0.01, *** *p* < 0.001; BMI = body mass index; MET = metabolic equivalent of task.

## Data Availability

All the data are presented in this study.
